# Supplementary material for: Comparison of fecal and blood metabolome reveals inconsistent associations of the gut microbiota with cardiometabolic diseases
Source: Nat Commun. 2023 Feb 2;14:571. doi: 10.1038/s41467-023-36256-y (PMC9894915; doi:10.1038/s41467-023-36256-y)
Supplement: Supplementary file 1 — Supplementary information [file 41467_2023_36256_MOESM1_ESM.pdf]

Supplementary Information

Comparison of fecal and blood metabolome reveals inconsistent associations of the gut microbiota with cardiometabolic diseases

This supplementary file contains the following:

Supplementary Notes 1:

Performance comparison between the RF and LightGBM model

Supplementary Figures:

Supplementary Fig. 1. Performance comparison of the RF and LightGBM model.

Supplementary Fig. 2. Phenotypic and genetic correlations between paired fecal and blood metabolites for (a) top 31-81 metabolites; (b) top 82-132 metabolites that are ranked by phenotypic correlations.

Supplementary Fig. 3. Phenotypic correlations between paired fecal and blood metabolites using partial *Spearman* correlation analysis, adjusted for age and sex versus adjusted for age, sex, and BMI.

Supplementary Fig. 4. Comparisons between paired fecal and blood metabolites in their associations with microbial pathways.

Supplementary Fig. 5. Comparisons between paired fecal and blood metabolites in their associations with taxonomic composition/microbial pathways.

Supplementary Fig. 6. Sensitivity analysis for gut microbiota-fecal/blood metabolite associations among participants (a) without T2D medications (n = 923); (b) without

hypertension medications (n = 706); (c) without dyslipidemia medications (n = 749); or (d) without any of the above three medications (n = 530), respectively.

Supplementary Fig. 7. Differences between the associations of gut microbiota with paired fecal and blood metabolites.

Supplementary Fig. 8. Sensitivity analysis for the identified associations between well-predicted fecal metabolites and prevalent cardiometabolic diseases, with an additional adjustment of T2D, hypertension, and dyslipidemia medications.

Supplementary Fig. 9. The associations between taxonomic composition/microbial pathways and well-predicted fecal/blood metabolites in the GNHS (discovery) and validation cohorts.

Supplementary Fig. 10. Comparisons between the associations of microbial pathways with paired fecal and blood SCFAs.

Supplementary Notes 1

Performance comparison between the RF and LightGBM model

The random forest (RF) and the Light Gradient Boosting Machine (LightGBM) models were commonly used machine learning pipelines to predict the metabolite levels based on gut microbiome [1, 2]. In this study, we used both of them to predict the concentrations of fecal/blood metabolites based on the taxonomic composition/microbial pathways and compared their performances. We first computed the root mean square error (RMSE) for each model that predict each metabolite levels based on taxonomic composition or microbial pathways. The RF model had significantly lower RMSE than the LightGBM model (0.967 ± 0.065 *vs.*0.975 ± 0.071; $P < 0.0001$; Supplementary Fig. 1a). Supplementary Fig. 1b showed that there was a strong correlation between the predictability results of metabolites obtained by the RF and LightGBM model (r = 0.994, $P < 0.0001$). We then defined metabolites with correlation coefficient > 0.3 and FDR < 0.05 as well-predicted metabolites. Supplementary Fig. 1c showed that there were significantly more well-predicted fecal metabolites than well-predicted blood metabolites based on either taxonomic composition or microbial pathways using both methods. There were more well-predicted metabolites obtained by the RF model than those obtained by the LightGBM model, especially for fecal metabolites based on microbial pathways (Supplementary Fig. 1c). Additionally, there was a large overlap between the taxonomic composition/microbial pathways-fecal/blood metabolite associations obtained by the RF and LightGBM model (Supplementary Fig. 1d). Besides the overlapped associations, we could obtain additional 10 significant

associations using the RF model, while there was only one additional association that was obtained using the LightGBM model (Supplementary Fig. 1d). Taken together, our results showed that the RF model had a better performance than the LightGBM model.

Supplementary Figures

Supplementary Fig. 1. Performance comparison of the RF and LightGBM model. (a) The distributions of the root mean square error (RMSE) for the RF and LightGBM model (n = 528). The difference between their distributions is tested by Wilcoxon signed-rank test. Box plots indicate median and interquartile range (IQR). The upper and lower whiskers indicate 1.5 times the IQR from above the upper quartile and below the lower quartile. *** $P < 0.0001$. **(b)** The scatter plot demonstrates the predictability results of metabolites obtained by the RF (*x* axis) and LightGBM (*y* axis) model. The correlation between the predictability results of metabolites obtained by the RF and LightGBM model is computed by *Pearson* correlation. **(c)** The number of well-predicted fecal and blood metabolites based on taxonomic composition or microbial pathways using the RF and LightGBM model. Well-predicted metabolites are defined as *Spearman*'s correlation coefficient > 0.3 and FDR < 0.05. FDR is controlled by the Benjamini-Hochberg method. **(d)** The overlap between taxonomic composition/microbial pathways-fecal/blood metabolite associations obtained by the RF and LightGBM model. All statistical tests are two-sided. Source data are provided as a Source Data file. RMSE, root mean square error; RF, random forest; LightGBM, Light Gradient Boosting Machine; FM, fecal metabolites; BM, blood metabolites; TC, taxonomic composition.

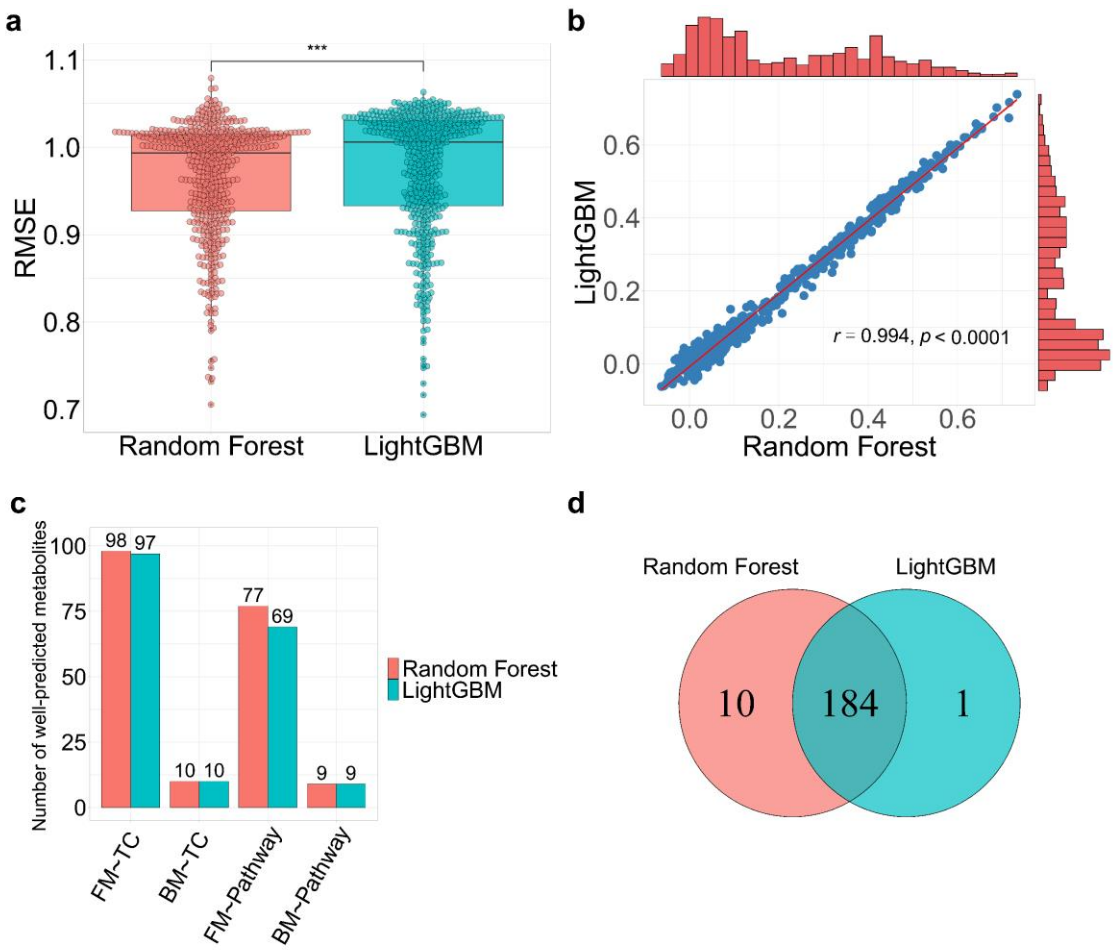
a
***
RMSE
1.1
1.0
0.9
0.8
0.7
Random Forest
LightGBM
b
LightGBM
0.6
0.4
0.2
0.0
r = 0.994, p < 0.0001
Random Forest
c
Number of well-predicted metabolites
100
75
50
25
0
98
97
10
10
77
69
9
9
Random Forest
LightGBM
FM~TC
BM~TC
FM~Pathway
BM~Pathway
d
Random Forest
LightGBM
10
184
1

Supplementary Fig. 2. Phenotypic and genetic correlations between paired fecal and blood metabolites for (a) top 31-81 metabolites; (b) top 82-132 metabolites that are ranked by phenotypic correlations. Phenotypic correlations between paired fecal and blood metabolites are estimated by partial *Spearman* correlation analysis, adjusted by age, sex and BMI. Genetic correlations are calculated using bivariate GREML analysis. Correlations with FDR < 0.05 and $|r|$ > 0.3 (red dashed lines) are considered significant. FDR is controlled by the Benjamini-Hochberg method. *FDR < 0.05, **FDR < 0.01, *** FDR < 0.005. All statistical tests are two-sided. Source data are provided as a Source Data file.

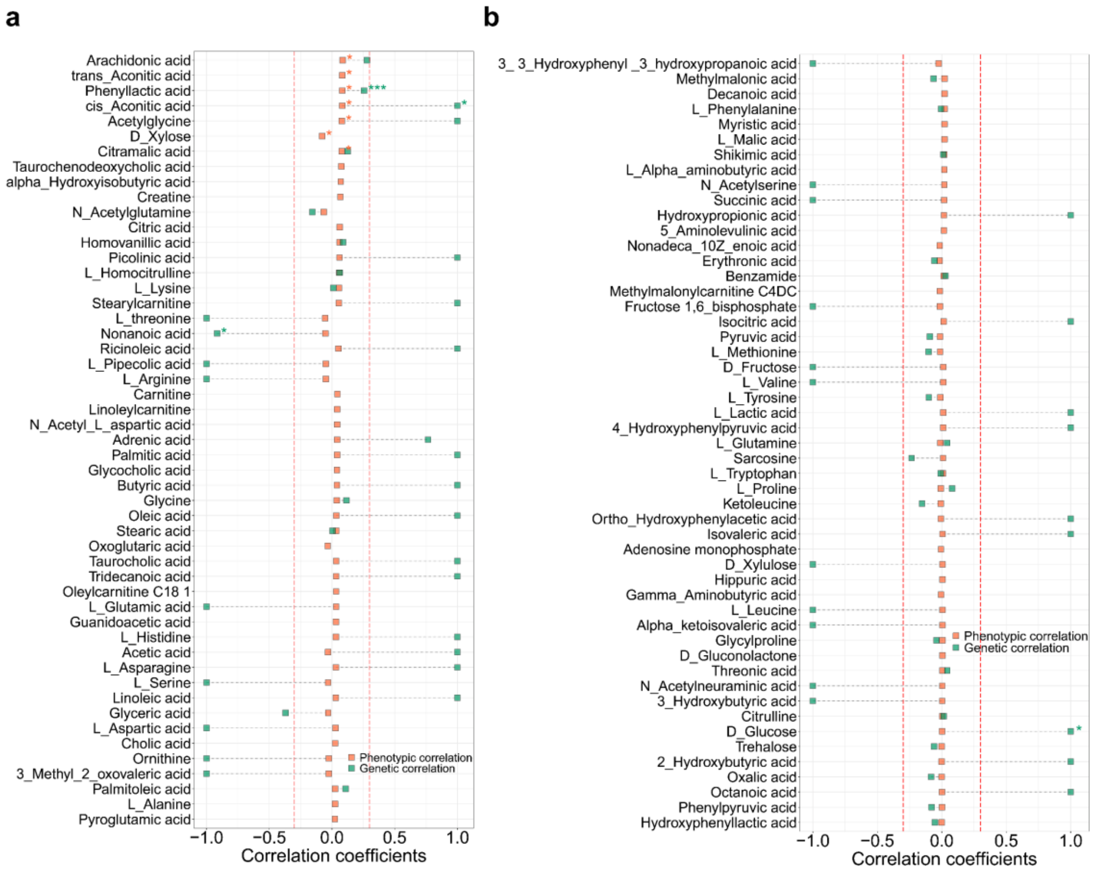

Supplementary Fig. 3. Phenotypic correlations between paired fecal and blood metabolites using partial *Spearman* correlation analysis, adjusted for age and sex versus adjusted for age, sex, and BMI. Correlation between phenotypic correlations with and without additional adjustment for BMI is calculated by *Pearson* correlation. All statistical tests are two-sided. Source data are provided as a Source Data file.

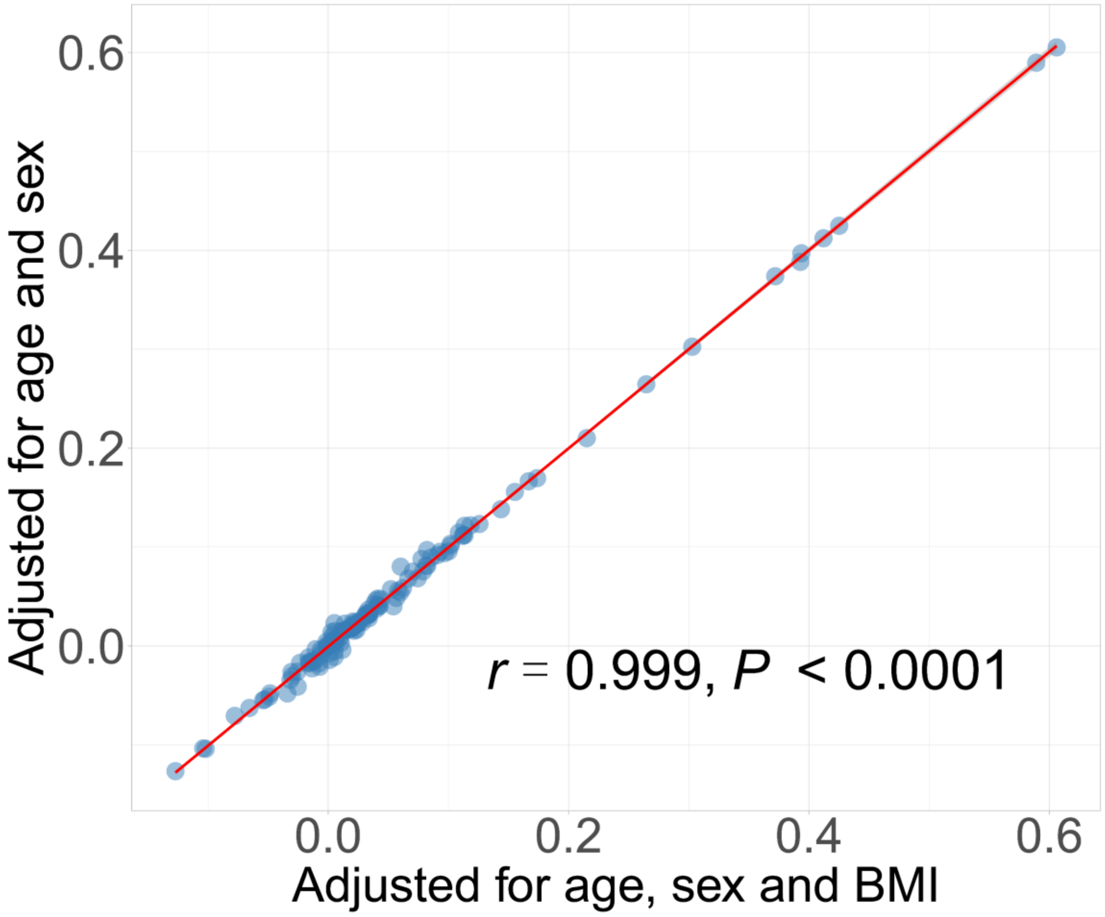

Supplementary Fig. 4. Comparisons between paired fecal and blood metabolites in their associations with microbial pathways. (a) The associations of microbial pathways with fecal metabolites; **(b)** with blood metabolites. The random forest model with five-fold cross-validation is used to predict the fecal or blood metabolite levels based on microbial pathways. *Spearman*'s correlation between measured and predicted metabolite levels is used to measure the association of microbial pathways with fecal or blood metabolites. *FDR < 0.05, **FDR < 0.01, *** FDR < 0.005. **(c)** The distributions of the associations of microbial pathways with fecal and blood metabolites (n = 132). The difference between the distributions of microbial pathways-fecal metabolite associations and microbial pathways-blood metabolite associations is tested by Wilcoxon signed-rank test. Box plots indicate median and interquartile range (IQR). The upper and lower whiskers indicate 1.5 times the IQR from above the upper quartile and below the lower quartile. *** $P < 0.0001$. **(d)** Differences between the associations of microbial pathways with paired fecal and blood metabolites that are well-predicted in both feces and blood (marked with red in y axis), metabolites that are only well-predicted in blood and not in feces (marked with green in y axis), and the top-30 metabolites that are only well-predicted in feces and not in blood (marked with blue in y axis). Metabolites are ranked by the predictability of fecal metabolites. Differences between the associations of taxonomic composition/microbial pathways with paired fecal and blood metabolites are tested by the method proposed by Hittner *et al*. (see Methods). *FDR < 0.05, **FDR < 0.01, *** FDR < 0.005. The results for the top 31-70 metabolites that are only well-

predicted in feces and not in blood are presented in Supplementary Fig. 7b. **(e)** The number of well-predicted fecal and blood metabolites based on microbial pathways and the number of validated associations between microbial pathways and well-predicted fecal/blood metabolites in the validation cohort. Well-predicted metabolites are defined as *Spearman*'s correlation coefficient > 0.3 and FDR < 0.05. FDR is controlled by the Benjamini-Hochberg method. Associations with *Spearman*'s correlation coefficient > 0.3 and FDR < 0.05 are considered as being validated in the validation cohort. All statistical tests are two-sided. Source data are provided as a Source Data file.

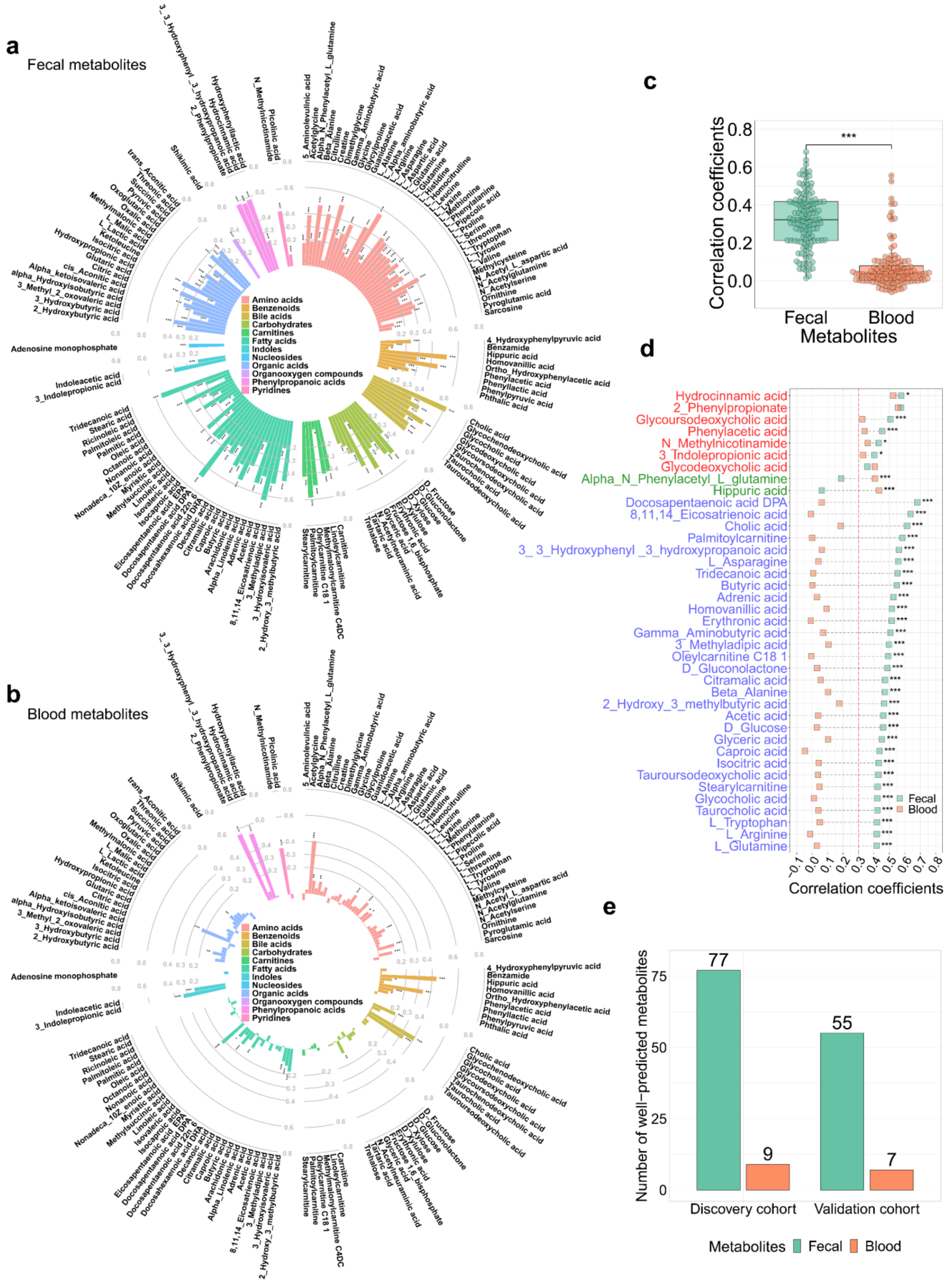
a
Fecal metabolites
b
Blood metabolites
Amino acids
Benzenoids
Bile acids
Carbohydrates
Carnitines
Fatty acids
Indoles
Nucleosides
Organic acids
Organooxygen compounds
Phenylpropanoic acids
Pyridines
c
Correlation coefficients
***
Fecal
Blood
Metabolites
d
Hydrocinnamic acid
2_Phenylpropionate
Glycoursodeoxycholic acid
Phenylacetic acid
N_Methylnicotinamide
3_Indolepropionic acid
Glycodeoxycholic acid
Alpha_N_Phenylacetyl_L_glutamine
Hippuric acid
Docosapentaenoic acid DPA
8,11,14_Eicosatrienoic acid
Cholic acid
Palmitoylcarnitine
3_3_Hydroxyphenyl_3_hydroxypropanoic acid
L_Asparagine
Tridecanoic acid
Butyric acid
Adrenic acid
Homovanillic acid
Erythronic acid
Gamma_Aminobutyric acid
3_Methyladipic acid
Oleylcarnitine C18 1
D_Gluconolactone
Citramalic acid
Beta_Alanine
2_Hydroxy_3_methylbutyric acid
Acetic acid
D_Glucose
Glyceric acid
Caproic acid
Isocitric acid
Tauroursodeoxycholic acid
Stearylcarnitine
Glycocholic acid
Taurocholic acid
L_Tryptophan
L_Arginine
L_Glutamine
Fecal
Blood
Correlation coefficients
e
Number of well-predicted metabolites
77
9
55
7
Discovery cohort
Validation cohort
Metabolites
Fecal
Blood

Supplementary Fig. 5. Comparisons between paired fecal and blood metabolites in their associations with taxonomic composition/microbial pathways. (a) The overlap between well-predicted metabolites based on taxonomic composition and microbial pathways for fecal metabolites and **(b)** for blood metabolites. **(c)** Sensitivity analysis for the number of well-predicted fecal and blood metabolites based on taxonomic composition or microbial pathways by setting the cut-off *r* value as 0.2. **(d)** by setting the cut-off *r* value as 0.4.

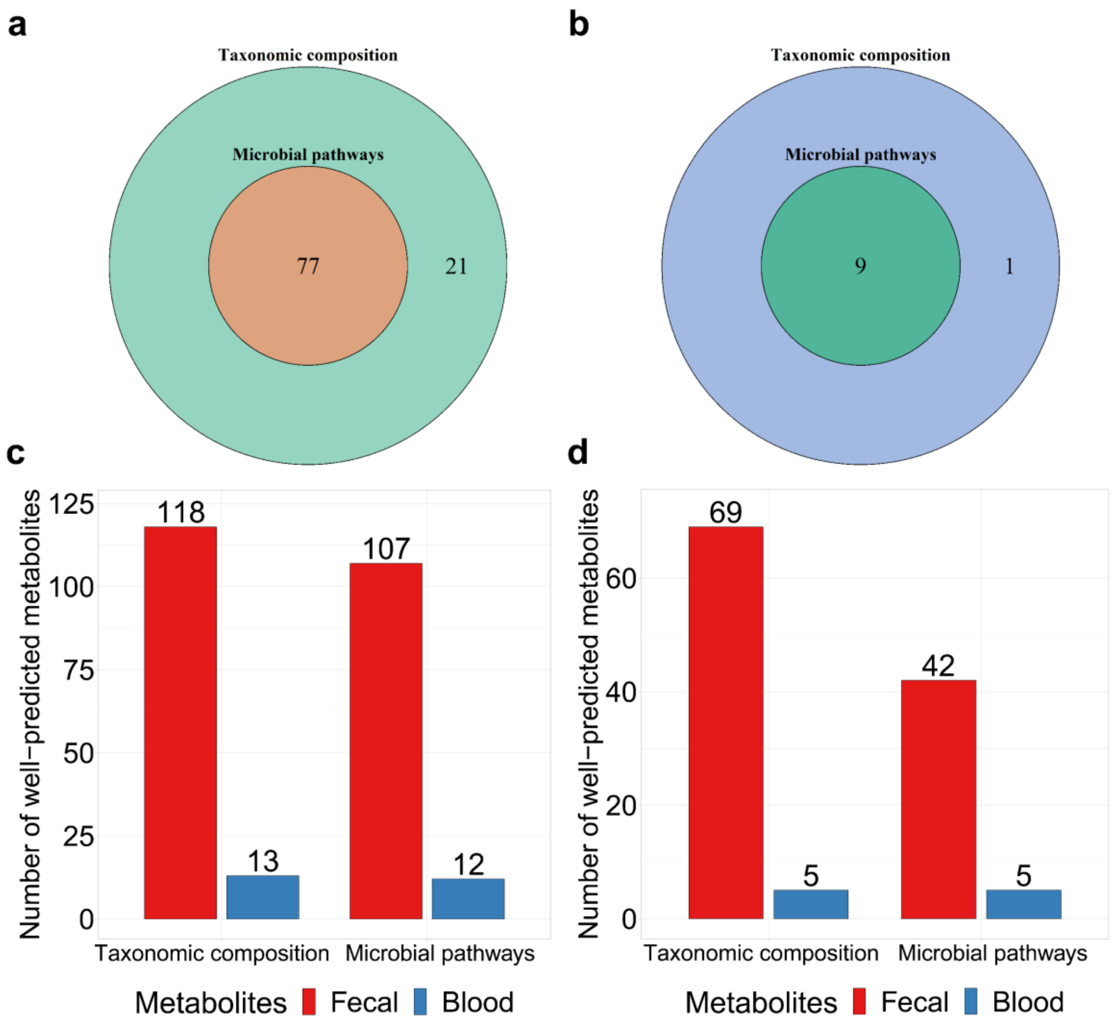

Supplementary Fig. 6. Sensitivity analysis for gut microbiota-fecal/blood metabolite associations among participants (a) without T2D medications (*n* = 923); (b) without hypertension medications (*n* = 706); (c) without dyslipidemia medications (*n* = 749); or (d) without any of the above three medications (*n* = 530), respectively. Correlation between gut microbiota-fecal/blood metabolite associations for all participants and those without medications is calculated by *Pearson* correlation. All statistical tests are two-sided. Source data are provided as a Source Data file. T2D, type 2 diabetes.

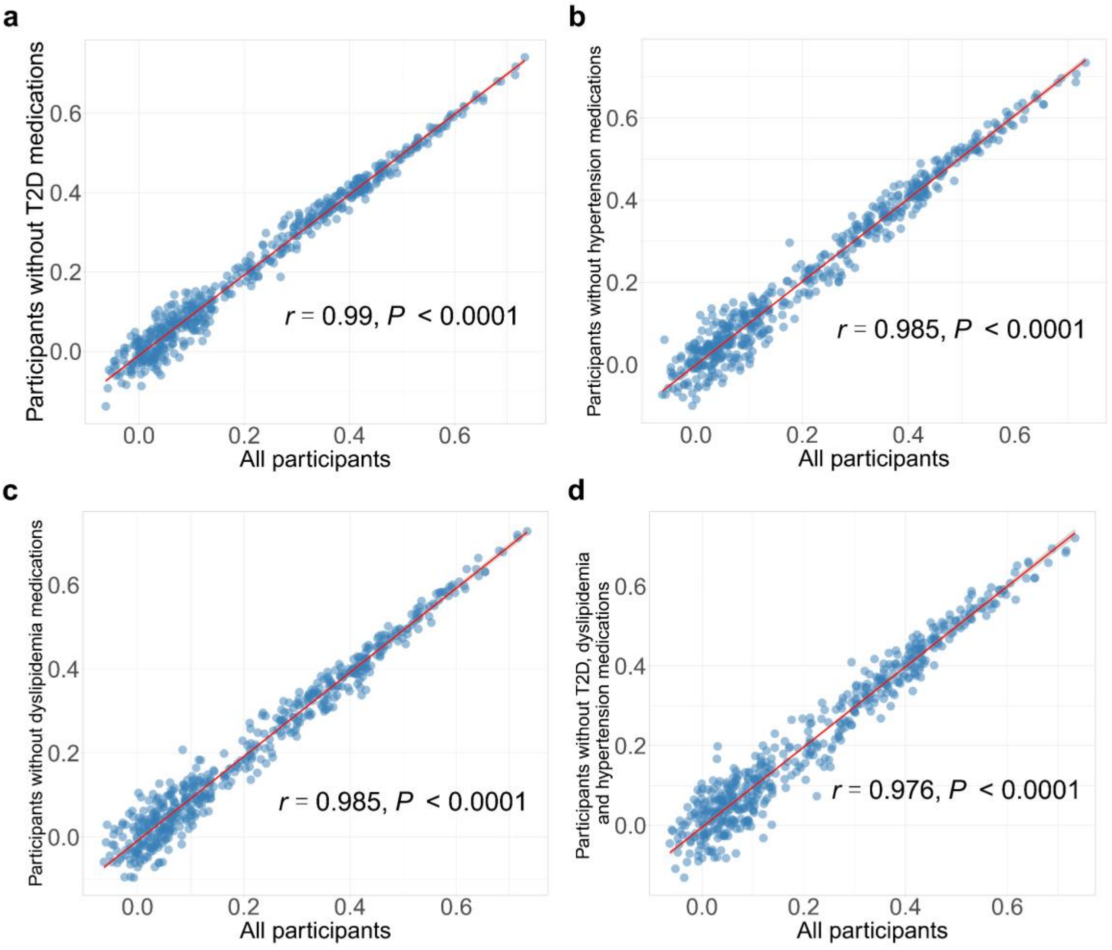

Supplementary Fig. 7. Differences between the associations of gut microbiota with paired fecal and blood metabolites. (a) Differences between the associations of taxonomic composition with paired fecal and blood metabolites for top 31-90 metabolites that are only well-predicted in feces and not in blood and are ranked by the predictability of fecal metabolites. **(b)** Differences between the associations of microbial pathways with paired fecal and blood metabolites for top 31-70 metabolites that are only well-predicted in feces and not in blood and are ranked by the predictability of fecal metabolites. Differences between the associations of taxonomic composition/microbial pathways with paired fecal and blood metabolites are tested by the method proposed by Hittner *et al.* (see Methods). FDR is controlled by the Benjamini-Hochberg method. *FDR < 0.05, **FDR < 0.01, *** FDR < 0.005. All statistical tests are two-sided. Source data are provided as a Source Data file.

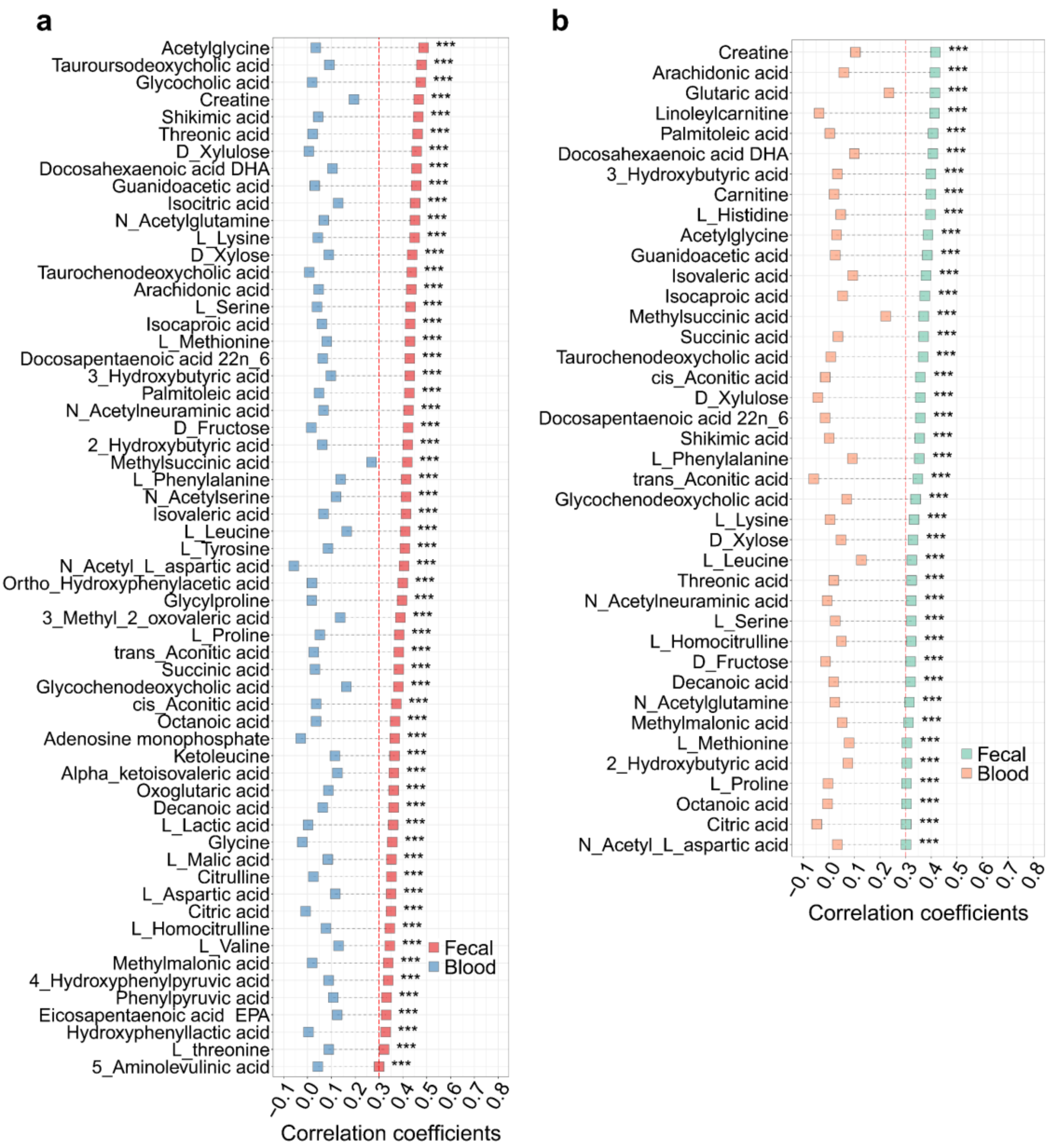
a
Acetylglycine
Tauroursodeoxycholic acid
Glycocholic acid
Creatine
Shikimic acid
Threonic acid
D_Xylulose
Docosahexaenoic acid DHA
Guanidoacetic acid
Isocitric acid
N_Acetylglutamine
L_Lysine
D_Xylose
Taurochenodeoxycholic acid
Arachidonic acid
L_Serine
Isocaproic acid
L_Methionine
Docosapentaenoic acid 22n_6
3_Hydroxybutyric acid
Palmitoleic acid
N_Acetylneuraminic acid
D_Fructose
2_Hydroxybutyric acid
Methylsuccinic acid
L_Phenylalanine
N_Acetylserine
Isovaleric acid
L_Leucine
L_Tyrosine
N_Acetyl_L_aspartic acid
Ortho_Hydroxyphenylacetic acid
Glycylproline
3_Methyl_2_oxovaleric acid
L_Proline
trans_Aconitic acid
Succinic acid
Glycochenodeoxycholic acid
cis_Aconitic acid
Octanoic acid
Adenosine monophosphate
Ketoleucine
Alpha_ketoisovaleric acid
Oxoglutaric acid
Decanoic acid
L_Lactic acid
Glycine
L_Malic acid
Citrulline
L_Aspartic acid
Citric acid
L_Homocitrulline
L_Valine
Methylmalonic acid
4_Hydroxyphenylpyruvic acid
Phenylpyruvic acid
Eicosapentaenoic acid EPA
Hydroxyphenyllactic acid
L_threonine
5_Aminolevulinic acid
Fecal
Blood
-0.1 0.0 0.1 0.2 0.3 0.4 0.5 0.6 0.7 0.8
Correlation coefficients
b
Creatine
Arachidonic acid
Glutaric acid
Linoleylcarnitine
Palmitoleic acid
Docosahexaenoic acid DHA
3_Hydroxybutyric acid
Carnitine
L_Histidine
Acetylglycine
Guanidoacetic acid
Isovaleric acid
Isocaproic acid
Methylsuccinic acid
Succinic acid
Taurochenodeoxycholic acid
cis_Aconitic acid
D_Xylulose
Docosapentaenoic acid 22n_6
Shikimic acid
L_Phenylalanine
trans_Aconitic acid
Glycochenodeoxycholic acid
L_Lysine
D_Xylose
L_Leucine
Threonic acid
N_Acetylneuraminic acid
L_Serine
L_Homocitrulline
D_Fructose
Decanoic acid
N_Acetylglutamine
Methylmalonic acid
L_Methionine
2_Hydroxybutyric acid
L_Proline
Octanoic acid
Citric acid
N_Acetyl_L_aspartic acid
Fecal
Blood
-0.1 0.0 0.1 0.2 0.3 0.4 0.5 0.6 0.7 0.8
Correlation coefficients

Supplementary Fig. 8. Sensitivity analysis for the identified associations between well-predicted fecal metabolites and prevalent cardiometabolic diseases, with an additional adjustment of T2D, hypertension, and dyslipidemia medications. Correlation between the associations of well-predicted fecal metabolites with prevalent cardiometabolic diseases with and without an additional adjustment of T2D, hypertension, and dyslipidemia medications is calculated by *Pearson* correlation. Error band is linear regression line with 95% confidence band. All statistical tests are two-sided. Source data are provided as a Source Data file. T2D, type 2 diabetes.

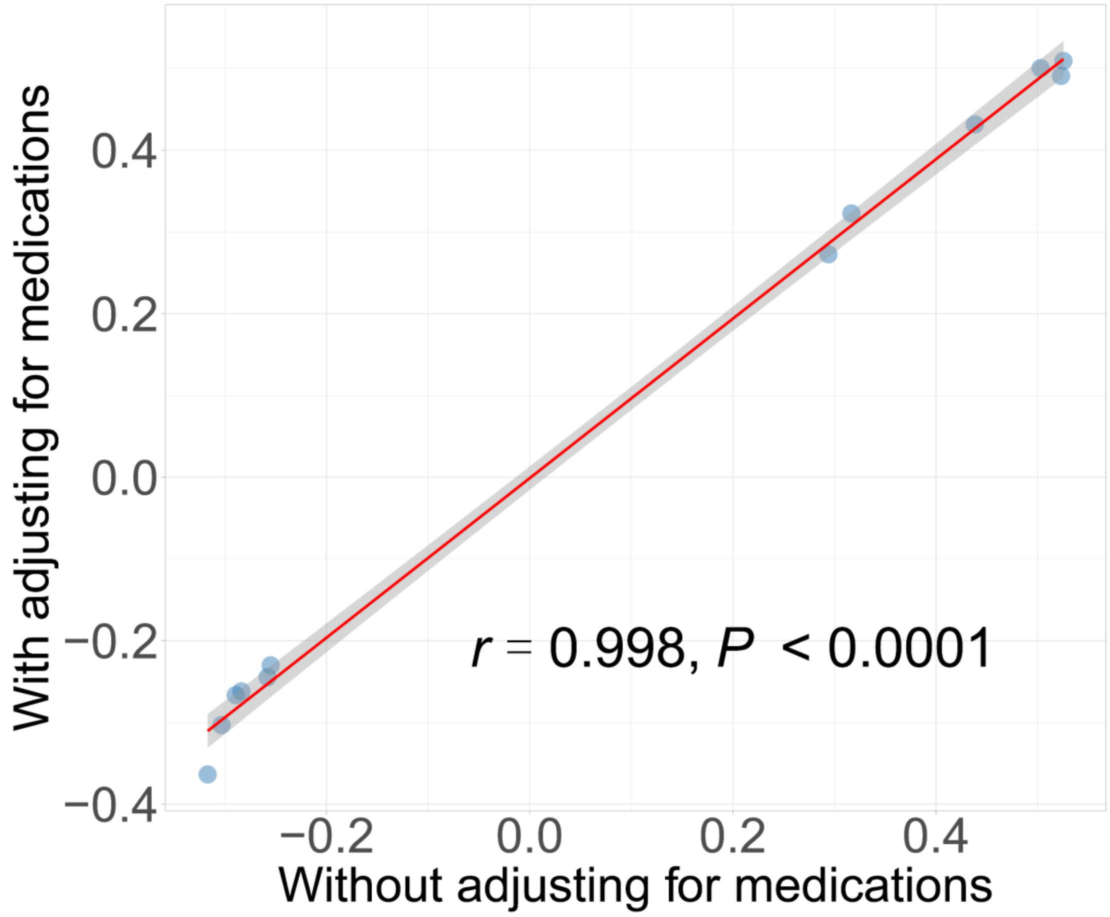

Supplementary Fig. 9. The associations between taxonomic composition/microbial pathways and well-predicted fecal/blood metabolites in the GNHS (discovery) and validation cohorts. (a) The associations between taxonomic composition and well-predicted fecal metabolites in the GNHS and validation cohort. **(b)** The associations between taxonomic composition and well-predicted blood metabolites in the GNHS and validation cohort **(c)** The associations between microbial pathways and well-predicted fecal metabolites in the GNHS and validation cohort. **(d)** The associations between microbial pathways and well-predicted blood metabolites in the GNHS and validation cohort. The associations between taxonomic composition/microbial pathways and well-predicted fecal/blood metabolites are measured by *Spearman*'s correlation between measured and predicted metabolite levels obtained by RF model. Only validated taxonomic composition/microbial pathways-fecal/blood metabolite associations are presented. Associations with *Spearman*'s correlation coefficient > 0.3 and FDR < 0.05 are considered as being validated in the validation cohort. FDR is controlled by the Benjamini-Hochberg method. All statistical tests are two-sided. Source data are provided as a Source Data file.

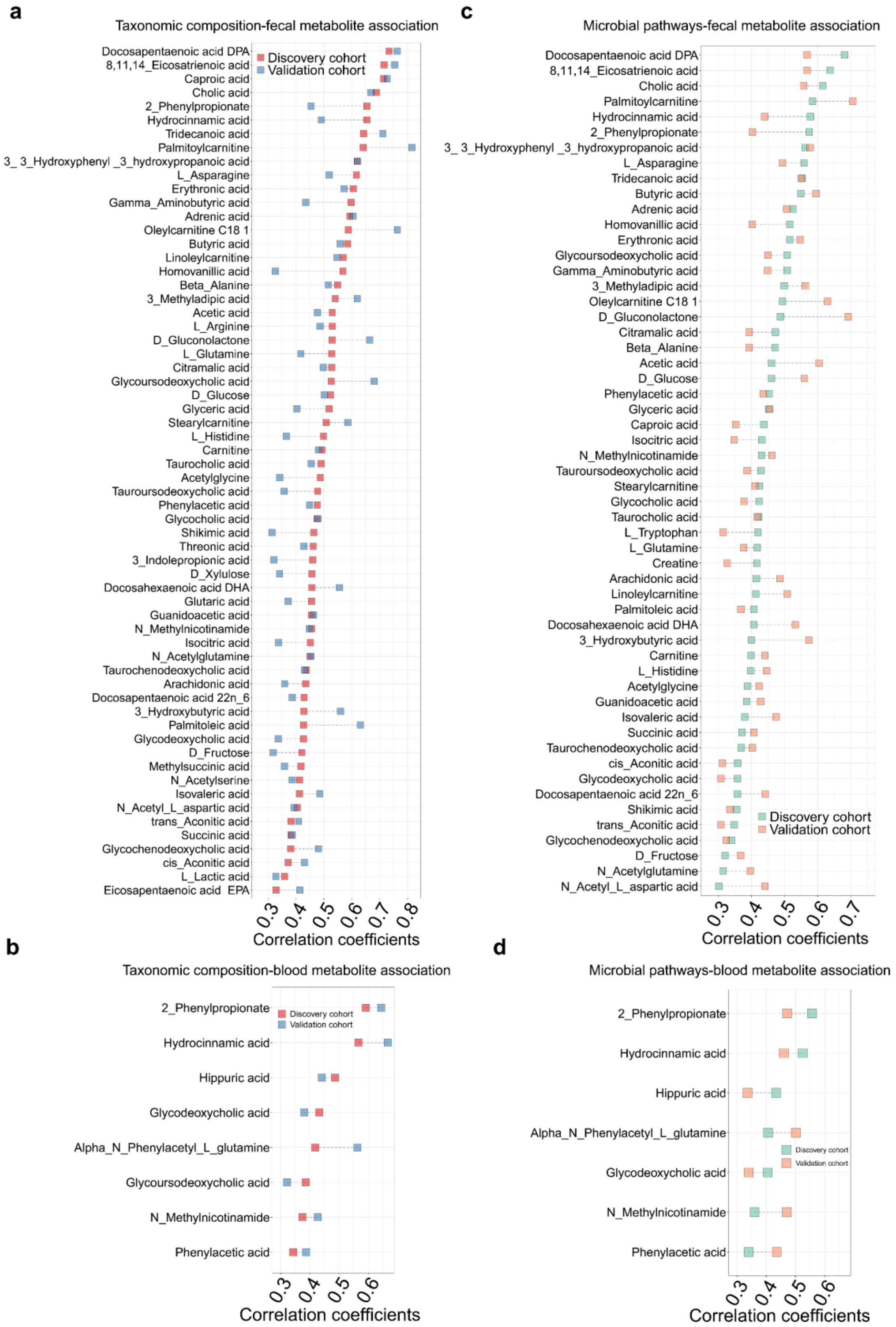
a
Taxonomic composition-fecal metabolite association
Discovery cohort
Validation cohort
Docosapentaenoic acid DPA
8,11,14_Eicosatrienoic acid
Caproic acid
Cholic acid
2_Phenylpropionate
Hydrocinnamic acid
Tridecanoic acid
Palmitoylcarnitine
3_3_Hydroxyphenyl _3_hydroxypropanoic acid
L_Asparagine
Erythronic acid
Gamma_Aminobutyric acid
Adrenic acid
Oleylcarnitine C18 1
Butyric acid
Linoleylcarnitine
Homovanillic acid
Beta_Alanine
3_Methyladipic acid
Acetic acid
L_Arginine
D_Gluconolactone
L_Glutamine
Citramalic acid
Glycoursodeoxycholic acid
D_Glucose
Glyceric acid
Stearylcarnitine
L_Histidine
Carnitine
Taurocholic acid
Acetylglycine
Tauroursodeoxycholic acid
Phenylacetic acid
Glycocholic acid
Shikimic acid
Threonic acid
3_Indolepropionic acid
D_Xylulose
Docosahexaenoic acid DHA
Glutaric acid
Guanidoacetic acid
N_Methylnicotinamide
Isocitric acid
N_Acetylglutamine
Taurochenodeoxycholic acid
Arachidonic acid
Docosapentaenoic acid 22n_6
3_Hydroxybutyric acid
Palmitoleic acid
Glycodeoxycholic acid
D_Fructose
Methylsuccinic acid
N_Acetylserine
Isovaleric acid
N_Acetyl_L_aspartic acid
trans_Aconitic acid
Succinic acid
Glycochenodeoxycholic acid
cis_Aconitic acid
L_Lactic acid
Eicosapentaenoic acid EPA
0.3 0.4 0.5 0.6 0.7 0.8
Correlation coefficients
c
Microbial pathways-fecal metabolite association
Docosapentaenoic acid DPA
8,11,14_Eicosatrienoic acid
Cholic acid
Palmitoylcarnitine
Hydrocinnamic acid
2_Phenylpropionate
3_3_Hydroxyphenyl _3_hydroxypropanoic acid
L_Asparagine
Tridecanoic acid
Butyric acid
Adrenic acid
Homovanillic acid
Erythronic acid
Glycoursodeoxycholic acid
Gamma_Aminobutyric acid
3_Methyladipic acid
Oleylcarnitine C18 1
D_Gluconolactone
Citramalic acid
Beta_Alanine
Acetic acid
D_Glucose
Phenylacetic acid
Glyceric acid
Caproic acid
Isocitric acid
N_Methylnicotinamide
Tauroursodeoxycholic acid
Stearylcarnitine
Glycocholic acid
Taurocholic acid
L_Tryptophan
L_Glutamine
Creatine
Arachidonic acid
Linoleylcarnitine
Palmitoleic acid
Docosahexaenoic acid DHA
3_Hydroxybutyric acid
Carnitine
L_Histidine
Acetylglycine
Guanidoacetic acid
Isovaleric acid
Succinic acid
Taurochenodeoxycholic acid
cis_Aconitic acid
Glycodeoxycholic acid
Docosapentaenoic acid 22n_6
Shikimic acid
trans_Aconitic acid
Glycochenodeoxycholic acid
D_Fructose
N_Acetylglutamine
N_Acetyl_L_aspartic acid
Discovery cohort
Validation cohort
0.3 0.4 0.5 0.6 0.7
Correlation coefficients
b
Taxonomic composition-blood metabolite association
2_Phenylpropionate
Discovery cohort
Validation cohort
Hydrocinnamic acid
Hippuric acid
Glycodeoxycholic acid
Alpha_N_Phenylacetyl_L_glutamine
Glycoursodeoxycholic acid
N_Methylnicotinamide
Phenylacetic acid
0.3 0.4 0.5 0.6
Correlation coefficients
d
Microbial pathways-blood metabolite association
2_Phenylpropionate
Hydrocinnamic acid
Hippuric acid
Alpha_N_Phenylacetyl_L_glutamine
Discovery cohort
Validation cohort
Glycodeoxycholic acid
N_Methylnicotinamide
Phenylacetic acid
0.3 0.4 0.5 0.6
Correlation coefficients

Supplementary Fig. 10. Comparisons between the associations of microbial pathways with paired fecal and blood SCFAs. (a) The association between microbial pathways and fecal acetic acid. **(b)** The association between microbial pathways and blood acetic acid. **(c)** The association between microbial pathways and fecal butyric acid. **(d)** The association between microbial pathways and blood butyric acid. The random forest model with five-fold cross-validation is used to predict the fecal or blood metabolite levels based on microbial pathways. The scatterplot is plotted by the predicted and measured metabolite values. *Spearman*'s correlation between measured and predicted metabolite values is used to measure the association of microbial pathways with fecal or blood metabolites. FDR is controlled by the Benjamini-Hochberg method. Error bands are linear regression lines with 95% confidence bands. All statistical tests are two-sided. Source data are provided as a Source Data file. FDR, false discovery rate.

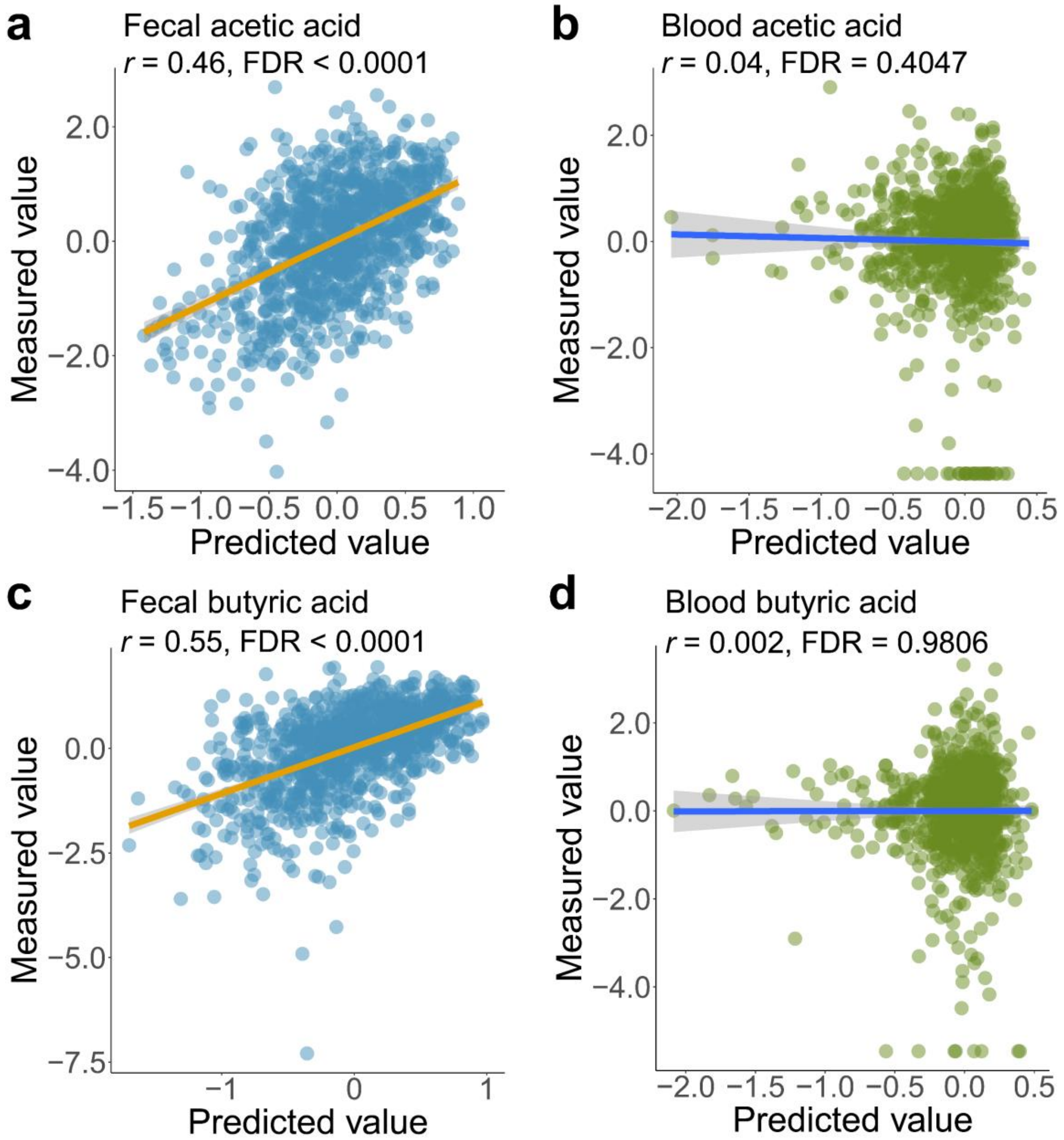
a
Fecal acetic acid
r = 0.46, FDR < 0.0001
Measured value
Predicted value
b
Blood acetic acid
r = 0.04, FDR = 0.4047
Measured value
Predicted value
c
Fecal butyric acid
r = 0.55, FDR < 0.0001
Measured value
Predicted value
d
Blood butyric acid
r = 0.002, FDR = 0.9806
Measured value
Predicted value

References

1. Muller E, Algavi YM, Borenstein E. A meta-analysis study of the robustness and universality of gut microbiome-metabolome associations. *Microbiome* **9**, 203 (2021).

2. Bar N*, et al.* A reference map of potential determinants for the human serum metabolome. *Nature* **588**, 135-140 (2020).
