# Supplementary material for: Comparison of fecal and blood metabolome reveals inconsistent associations of the gut microbiota with cardiometabolic diseases
Source: Nat Commun. 2023 Feb 2;14:571. doi: 10.1038/s41467-023-36256-y (PMC9894915; doi:10.1038/s41467-023-36256-y)
Supplement: Supplementary file 3 — Description of Additional Supplementary Files [file 41467_2023_36256_MOESM3_ESM.docx]

**Description of Additional Supplementary Files**

**File Name: Supplementary Data 1**

**Description:** The results of phenotypic and genetic correlations, related to Fig. 2 and Supplementary Fig. 2. Phenotypic correlations between paired fecal and blood metabolites are estimated by partial Spearman correlation analysis, adjusted by age, sex and BMI. Genetic correlations are calculated using bivariate GREML analysis. FDR is controlled by the Benjamini-Hochberg method. All statistical tests are two-sided. FDR, false discovery rate.

**File Name: Supplementary Data 2**

**Description:** The predictability of fecal and blood metabolites based on taxonomic composition or microbial pathways using machine learning pipeline, related to Fig. 3 and Supplementary Fig. 4. The predictability is measured by Spearman’s correlation between the measured and predicted metabolite levels for held-out samples by random forest model with five-fold cross-validation. FDR is controlled by the Benjamini-Hochberg method. All statistical tests are two-sided. FDR, false discovery rate.

**File Name: Supplementary Data 3**

**Description:** Differences between the associations of taxonomic composition/microbial pathways with paired fecal and blood metabolites, related to Fig. 3, Supplementary Fig. 4, and Supplementary Fig. 7. Differences between the associations of taxonomic composition/microbial pathways with paired fecal and blood metabolites are tested by the method proposed by Hittner et al. (see Methods). FDR is controlled by the Benjamini-Hochberg method. All statistical tests are two-sided. FDR, false discovery rate.

**File Name: Supplementary Data 4**

**Description:** The associations of taxonomic composition/microbial pathways with well-predicted fecal and blood metabolites in the discovery and validation cohorts, related to Supplementary Fig. 9. The associations between taxonomic composition/microbial pathways and well-predicted fecal/blood metabolites are measured by Spearman’s correlation between measured and predicted metabolite levels obtained by random forest model. Only validated taxonomic composition/microbial pathways-fecal/blood metabolite associations are presented. Associations with Spearman’s correlation coefficient > 0.3 and FDR < 0.05 are considered as being validated in the validation cohort. FDR is controlled by the Benjamini-Hochberg method. All statistical tests are two-sided. GNHS, Guangzhou Nutrition and Health Study; FDR, false discovery rate.

**File Name: Supplementary Data 5**

**Description:** The associations of well-predicted fecal and blood metabolites with cardiometabolic diseases, related to Fig. 4. The associations of well-predicted fecal and blood metabolites with cardiometabolic diseases are estimated by logistic models, adjusted by age, sex, smoking status, alcohol status, education, income, physical activity, and total energy intake for obesity, and by age, sex, BMI, smoking status, alcohol status, education, income, physical activity, and total energy intake for T2D, hypertension and NAFLD. FDR is controlled by the Benjamini-Hochberg method. All statistical tests are two-sided. CI, confidence interval; OR, odds ratio; FDR, false discovery rate.
