# Supplementary material for: Comparison of fecal and blood metabolome reveals inconsistent associations of the gut microbiota with cardiometabolic diseases
Source: Nat Commun. 2023 Feb 2;14:571. doi: 10.1038/s41467-023-36256-y (PMC9894915; doi:10.1038/s41467-023-36256-y)
Supplement: Supplementary file 9 — Reporting Summary [file 41467_2023_36256_MOESM9_ESM.pdf]

Corresponding author(s): Yu-ming Chen and Ju-Sheng Zheng

Last updated by author(s): Jan 9, 2023

Reporting Summary

Nature Portfolio wishes to improve the reproducibility of the work that we publish. This form provides structure for consistency and transparency in reporting. For further information on Nature Portfolio policies, see our Editorial Policies and the Editorial Policy Checklist.

Statistics

For all statistical analyses, confirm that the following items are present in the figure legend, table legend, main text, or Methods section.

n/a	Confirmed	
☐	☒	The exact sample size (n) for each experimental group/condition, given as a discrete number and unit of measurement
☐	☒	A statement on whether measurements were taken from distinct samples or whether the same sample was measured repeatedly
☐	☒	The statistical test(s) used AND whether they are one- or two-sided<br>*Only common tests should be described solely by name; describe more complex techniques in the Methods section.*
☐	☒	A description of all covariates tested
☐	☒	A description of any assumptions or corrections, such as tests of normality and adjustment for multiple comparisons
☐	☒	A full description of the statistical parameters including central tendency (e.g. means) or other basic estimates (e.g. regression coefficient) AND variation (e.g. standard deviation) or associated estimates of uncertainty (e.g. confidence intervals)
☐	☒	For null hypothesis testing, the test statistic (e.g. F, t, r) with confidence intervals, effect sizes, degrees of freedom and P value noted<br>*Give P values as exact values whenever suitable.*
☒	☐	For Bayesian analysis, information on the choice of priors and Markov chain Monte Carlo settings
☒	☐	For hierarchical and complex designs, identification of the appropriate level for tests and full reporting of outcomes
☐	☒	Estimates of effect sizes (e.g. Cohen's d, Pearson's r), indicating how they were calculated

Our web collection on statistics for biologists contains articles on many of the points above.

Software and code

Policy information about availability of computer code

Data collection	No specific software was used for data collection.
Data analysis	All data analyses were conducted using publicly available tools. The following softwares were used in this study: GCTA (version v1.93.3); R software (version 4.1.1); R packages: randomForest (version: 4.6-14), lightgbm (version: 3.3.1), metafor (version: 3.0-2); QuanMET software (version 2.0); PRINSEQ (version 0.20.447); Bowtie2 (version 2.2.5); MetaPhlAn2 (version 2.6.02); and HUMAnN2 (version 2.8.1). Codes used for data analysis is publicly available at: https://github.com/nutrition-westlake/Paired-comparisons-between-the-fecal-and-blood-metabolites-in-their-associations-with-gut-microbiota/tree/main

For manuscripts utilizing custom algorithms or software that are central to the research but not yet described in published literature, software must be made available to editors and reviewers. We strongly encourage code deposition in a community repository (e.g. GitHub). See the Nature Portfolio guidelines for submitting code & software for further information.

Data

Policy information about availability of data

All manuscripts must include a data availability statement. This statement should provide the following information, where applicable:
- Accession codes, unique identifiers, or web links for publicly available datasets
- A description of any restrictions on data availability
- For clinical datasets or third party data, please ensure that the statement adheres to our policy

The raw data of metagenomic sequencing in this study have been deposited in the Genome Sequence Archive (GSA) (https://ngdc.cncb.ac.cn/gsa/) at accession

number CRA008796. The fecal and serum metabolomics data have been deposited in the Metabolomics Workbench at study ID ST002337 and ST001669, respectively. UCSC hg19 is available from https://ftp.ebi.ac.uk/pub/databases/gencode/Gencode_human/release_19/GRCh37.p13.genome.fa.gz. The data associated with this study are presented in the paper (Supplementary Table S1-S5 and Source Data). Source data are provided with this paper. The metadata are available under restricted access due to participant consent and privacy regulations of our cohort, access can be obtained by request to the corresponding author (Yu-ming Chen: chenyum@mail.sysu.edu.cn)

Human research participants

Policy information about studies involving human research participants and Sex and Gender in Research.

Reporting on sex and gender	This study includes both sexes. Sex was determined based on self-reporting. There were 695 females and 312 males in the discovery cohort , and 74 females and 29 males in the validation cohort. Sex was included as a covariant for association analysis, and no sex-specific analysis was performed.
Population characteristics	Our study was based on the Guangzhou Nutrition and Health Study (GNHS, discovery cohort, N=1007) and the control arm of a hip fraction case-control study (Validation cohort, N=103), who had paired fecal and blood samples (Table 1).
Recruitment	The GNHS is a community-based prospective study, in which Chinese participants aged 40-75 years living in the urban area of Guangzhou, China, for at least 5 years were recruited between 2008-2013. In the control arm of a hip fraction case-control study, participants were enrolled between 2009 and 2012 in Guangdong Province, China.
Ethics oversight	The Ethics Committee of the School of Public Health at Sun Yat-sen University (2018048) and Westlake University (20190114ZJS0003) approved this study.

Note that full information on the approval of the study protocol must also be provided in the manuscript.

Field-specific reporting

Please select the one below that is the best fit for your research. If you are not sure, read the appropriate sections before making your selection.

[x] Life sciences [ ] Behavioural & social sciences [ ] Ecological, evolutionary & environmental sciences

For a reference copy of the document with all sections, see nature.com/documents/nr-reporting-summary-flat.pdf

Life sciences study design

All studies must disclose on these points even when the disclosure is negative.

Sample size	No sample size calculation was performed because the present study is observational, not interventional. We included participants with matched gut metagenomic, fecal and blood metabolomics data collected at the same time point. For this type of data, our study has the largest sample size in the world.
Data exclusions	In the GNHS, a total of 4,048 Chinese participants aged 40-75 years living in the urban area of Guangzhou, China, for at least 5 years were recruited between 2008-2013. We followed these participants every three years. During 2014-2018 follow-up visits, stool and fasting blood samples were collected at the same time. We included participants with paired fecal and blood samples (N=1008). We excluded participants who have taken antibiotics within 2 weeks (N=1). Finally, 1007 participants were remained for subsequent analysis.<br>In the control arm of a case-control study for hip fraction (validation cohort), participants were enrolled between 2009 and 2012 in Guangdong Province, China. Stool and fasting blood samples were collected at the follow-up visits between February 2017 and May 2017 at the same time point. We included 103 participants with paired fecal and blood samples in the present study.
Replication	68% gut microbiota-fecal/blood metabolite associations could be validated in the validation cohort.
Randomization	Not applicable for this observational study.
Blinding	Not applicable for this observational study.

Reporting for specific materials, systems and methods

We require information from authors about some types of materials, experimental systems and methods used in many studies. Here, indicate whether each material, system or method listed is relevant to your study. If you are not sure if a list item applies to your research, read the appropriate section before selecting a response.

Materials & experimental systems

n/a	Involved in the study
☒	☐ Antibodies
☒	☐ Eukaryotic cell lines
☒	☐ Palaeontology and archaeology
☒	☐ Animals and other organisms
☒	☐ Clinical data
☒	☐ Dual use research of concern

Methods

n/a	Involved in the study
☒	☐ ChIP-seq
☒	☐ Flow cytometry
☒	☐ MRI-based neuroimaging
